# Retraction

**DOI:** 10.1111/jcmm.17151

**Published:** 2022-01-10

Intestinal fatty acid‐binding protein mediates atherosclerotic progress through increasing intestinal inflammation and permeability. Zhang, L, Wang, F, Wang, J, Wang, Y, Fang, Y. *J Cell Mol Med*. 2020; 24: 5205– 5212. https://doi.org/10.1111/jcmm.15173

The above article, published online on 27 March 2020 on Wiley Online Library (wileyonlinelibrary.com), has been retracted by agreement among the journal's Editor‐in‐Chief, Stefan N. Constantinescu, the authors, the Foundation for Cellular and Molecular Medicine, and John Wiley & Sons Ltd. The retraction has been agreed following concerns raised about similarities between Figure 2E in the above article and Figure 2C–E in another published article.^1^ Upon further analysis of the images, it was determined that the images in Figure 2E in the above article had been taken without attribution from Figure 2C–E^1^ and manipulated (resized, cropped and inverted). As a result, the journal has concerns about the results and conclusions drawn and will retract the article.
